# Exploratory Analysis of ^18^F-3’-deoxy-3’-fluorothymidine (^18^F-FLT) PET/CT-Based Radiomics for the Early Evaluation of Response to Neoadjuvant Chemotherapy in Patients With Locally Advanced Breast Cancer

**DOI:** 10.3389/fonc.2021.601053

**Published:** 2021-06-24

**Authors:** Lorenzo Fantini, Maria Luisa Belli, Irene Azzali, Emiliano Loi, Andrea Bettinelli, Giacomo Feliciani, Emilio Mezzenga, Anna Fedeli, Silvia Asioli, Giovanni Paganelli, Anna Sarnelli, Federica Matteucci

**Affiliations:** ^1^ Nuclear Medicine Unit, IRCCS Istituto Romagnolo per lo Studio dei Tumori (IRST) “Dino Amadori,” Meldola, Italy; ^2^ Medical Physics Unit, IRCCS Istituto Romagnolo per lo Studio dei Tumori (IRST) “Dino Amadori,” Meldola, Italy; ^3^ Biostatistics and Clinical Trials Unit, IRCCS Istituto Romagnolo per lo Studio dei Tumori (IRST) “Dino Amadori,” Meldola, Italy; ^4^ Medical Physics Department, Veneto Institute of Oncology IOV - IRCCS, Padua, Italy; ^5^ Department of Medical Oncology, IRCCS Istituto Romagnolo per lo Studio dei Tumori (IRST) “Dino Amadori,” Meldola, Italy; ^6^ Pathology Unit, Morgagni-Pierantoni Hospital, Forlì, Italy

**Keywords:** radiomics, fluorothymidine positron emission tomography scan, breast cancer, early response, neoadjuvant chemotherapy

## Abstract

**Purpose:**

The objective of this study was to evaluate a set of radiomics-based advanced textural features extracted from ^18^F-FLT-PET/CT images to predict tumor response to neoadjuvant chemotherapy (NCT) in patients with locally advanced breast cancer (BC).

**Materials and Methods:**

Patients with operable (T2-T3, N0-N2, M0) or locally advanced (T4, N0-N2, M0) BC were enrolled. All patients underwent chemotherapy (six cycles every 3 weeks). Surgery was performed within 4 weeks of the end of NCT. The MD Anderson Residual Cancer Burden calculator was used to evaluate the pathological response. ^18^F-FLT-PET/CT was performed 2 weeks before the start of NCT and approximately 3 weeks after the first cycle. The evaluation of PET response was based on EORTC criteria. Standard uptake value (SUV) statistics (SUV_max_, SUV_peak_, SUV_mean_), together with 148 textural features, were extracted from each lesion. Indices that are robust against contour variability (ICC test) were used as independent variables to logistically model tumor response. LASSO analysis was used for variable selection.

**Results:**

Twenty patients were included in the study. Lesions from 15 patients were evaluable and analyzed: 9 with pathological complete response (pCR) and 6 with pathological partial response (pPR). Concordance between PET response and histological examination was found in 13/15 patients. LASSO logistic modelling identified a combination of SUV_max_ and the textural feature index IVH_VolumeIntFract_90 as the most useful to classify PET response, and a combination of PET response, ID range, and ID_Coefficient of Variation as the most useful to classify pathological response.

**Conclusions:**

Our study suggests the potential usefulness of FLT-PET for early monitoring of response to NCT. A model based on PET radiomic characteristics could have good discriminatory capacity of early response before the end of treatment.

## Introduction

Neoadjuvant chemotherapy (NCT) followed by surgery represents the standard strategy in the treatment of locally advanced breast cancer, obtaining an objective response rate of around 70% and a complete pathological response rate of up to 30% (1–3).

It is well known that the pathological response to NCT represents an important prognostic factor in this setting (4–6). However, the evaluation of the response is mainly based on the histopathological findings of the surgical sample. There is thus a clear need for an ongoing evaluation of chemotherapy to early differentiate between responders and non-responders, which would enable the latter to be offered alternative therapies, reduce the risk of chemotherapy-related toxicity, and lower costs for the National Health Service.

At present, there is no unanimous agreement about the best imaging method for the early assessment of response to therapy. Morphology-based imaging methods are generally used and interpreted by Response Evaluation Criteria in Solid Tumors criteria (RECIST v1.1) (7). However, given that morphological changes may arise late in the course of treatment, RECIST v1.1 criteria may not be able to identify response at an earlier stage. Existing evidence of changes in tumor morphology, likely preceded by changes in metabolism, has led to the use of functional imaging methods such as positron emission tomography (PET) for assessing early response to therapy.

Currently, ^18^F-labeled fluorodeoxyglucose (FDG) is the most widely used method to monitor response to therapy in BC. Although ^18^F-FDG is a sensitive tracer, it is not highly tumor-specific as it also accumulates in activated macrophages and other inflammatory cells, with a consequent increase in potential false-positive responses (8).

In the search for more specific tracers for BC, ^18^F-labeled fluorothymidine (^18^F-FLT) has been proposed as an indicator of proliferation (9) because thymidine is an analog of pyrimidine, which is incorporated in DNA but not in RNA. Thus, the possibility of quantifying the proliferative activity of the tumor through the use of ^18^F-FLT PET/CT could represent a potentially useful tool for evaluating the viability of tumor cells during or at the start of treatment.

The most widely used parameter to measure lesion uptake is the standard uptake value (SUV). Semi-quantitative SUV statistics (*e.g.* SUV_max_, SUV_peak_, SUV_mean_) are generally used to measure uptake inside a region. The potential utility of ^18^F-FLT PET/CT images as an early indicator of treatment response, in terms of SUV_max_, in patients undergoing NCT was previously demonstrated by Crippa et al. (10).

Although semi-quantitative SUV indices provide overall information on uptake, they are not capable of detecting the presence of non-uniform uptake distribution. However, it is known that tracer uptake within a tumor mass is characterized by significant heterogeneity because of various factors such as metabolism, hypoxia, necrosis, and cell proliferation (11). This heterogeneity appears to correlate with tumor aggressiveness, response to treatment, and prognosis (12).

Radiomics, an approach capable of quantifying the heterogeneity of textures in medical imaging, is an emerging translational research field that may be able to provide more accurate information than the semi-quantitative parameters normally used. The application of radiomic analysis capable of extracting textural features has been used for FDG PET/CT images, with good results (13–16). The availability of textural features before therapy or during the first treatment phases could thus facilitate decision-making in relation to the therapeutic strategy to adopt.

We evaluated a subgroup of patients enrolled in a multicenter phase II trial of liposomal doxorubicin, docetaxel, and trastuzumab in combination with metformin as NCT for HER2-positive BC. The main objective of the present study was to investigate the role of radiomics-based advanced imaging features extracted from ^18^F-FLT PET/CT images to predict tumor response to NCT in patients with BC.

## Materials and Methods

### Patients

We selected 20 patients with operable (T2-T3, N0-N2, M0) or locally advanced (T4, N0-N2, M0) human epidermal growth factor receptor 2-positive (HER2-positive) BC taking part in multicenter phase II trial at our cancer (IRST IRCCS).

All patients gave written consent to participate in the study, which was approved by the Local Research and Ethics Committee (Eudract no. 2014-002602-20; Protocol Code: IRST174.09; ClinicalTrials.gov NCT02488564).

**Figure 1:**
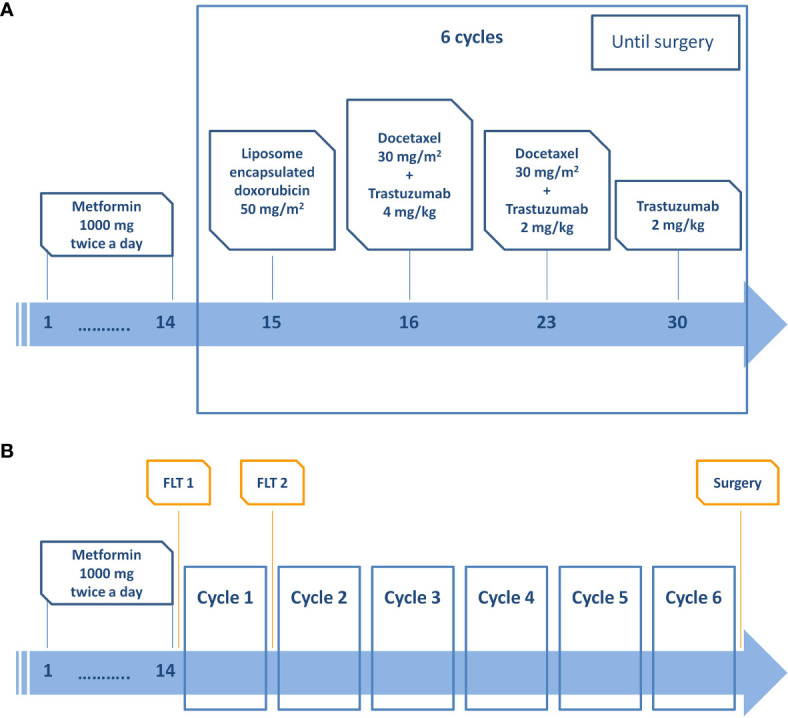
**(A)** Treatment scheme and timeline. **(B)** Image acquisition scheme and timeline.

Before surgery, all patients were submitted to chemotherapy with liposome-encapsulated doxorubicin (every 3 weeks, for six cycles) plus docetaxel (every 3 weeks, for six cycles) plus trastuzumab plus metformin (1,000 mg twice a day per os) (**Figure 1A**).

Surgery was performed within 2 to 4 weeks of the end of chemotherapy followed by radiotherapy to the residual breast level (for patients undergoing conservative surgery) or chest wall (T4 tumors).

After surgery, if deemed suitable by the investigator, patients were treated with adjuvant anthracycline/paclitaxel or with the cyclophosphamide, methotrexate, and 5-fluorouracil (CMF) scheme. Herceptin was administered every 3 weeks for 1 year.

### Imaging Protocols

^18^F-FLT was produced by Advanced Accelerator Applications (AAA) with a radiochemical purity and specific activity >95% and >1 Ci/μmol, respectively.

All patients underwent a ^18^F-FLT-PET/CT scan a maximum of 2 weeks before the start of NCT (FLT1, basal) and immediately before the second cycle of chemotherapy (FLT2) (**Figure 1B**). Patients were weighed before each scan and given an intravenous injection of 3.5 MBq/kg of FLT (maximum activity 350 MBq). No dietary restrictions were required before imaging. All patients were scanned in the supine position and were informed of the importance of remaining perfectly still and of maintaining shallow breathing throughout the procedure.

Images were acquired on two different PET/CT scanners. The first scanner was a Discovery LS (GE Healthcare, UK) equipped with 18 rings (92.7 cm diameter), each containing 672 BGO crystals (4 mm × 8 mm × 30 mm crystal size), 152 mm axial FOV dimension, combined with a four-slice CT system. Step-and-shoot mode was used for whole-body image acquisition. The second scanner was a Biograph mCT Flow 64-4R PET/CT (Siemens Healthcare, Germany) equipped with four rings (84.2 cm diameter) of 48 detector blocks, each containing 13 × 13 LSO crystals (4 mm × 4 mm × 20 mm crystal size), 221 mm axial FOV dimension, combined with a 64-slice CT system. Continuous table motion mode was used for whole-body image acquisition (17). Both point-spread-function and time-of-light corrections are available on this scanner. Both FLT1 and FLT2 were acquired on the same scanner for each patient.

Image acquisition started 1 h after intravenous injection of ^18^F-FLT: a CT was performed from the brain to the pelvis immediately before the PET scan, with a multidetector spiral CT scanner (Discovery LS: 3.9 × 3.9 mm^2^ pixel dimension, 5 mm slice thickness, pitch 1.75, 120 keV and 20–200 auto mA; Biograph: 0.98 × 0.98 mm^2^ pixel dimension, 3 mm slice thickness, pitch 1.2, 120 keV and auto mA [30–200 mA depending on the patient’s total body mass]). Whole-body PET scan was performed, covering an area identical to that covered by the CT. PET data were reconstructed into a 128 × 128 matrix (voxel dimension 3.9 × 3.9 and 4.1 × 4.1 mm^2^, slicethickness 4.25 and 3 mm, for Discovery LS and Biograph mCT Flow scanner, respectively), using the OSEM reconstruction algorithm. Corrections were applied for attenuation, scatter, random coincidences, isotope decay, and dead time. Fused PET and CT images were subsequently generated.

### Image Analysis

#### Volume Definition

All volumes of interest (VOIs) encompassing the lesions were manually contoured on both FLT1 and FLT2 CT images by an expert nuclear medicine radiologist (LF) blinded to patient outcome. A second senior nuclear medicine radiologist (FM) supervised each contour. The MimVista software (Mim Software Inc., v6.6, OH, USA) was used for PET/CT image rigid registration and lesion delineation.

In order to test the robustness of textural features against contour variability, four contours (original lesion VOI, expansion of +1 mm, +2 mm, contraction of −1 mm) were considered.

PET image and structure set were then extrapolated in DICOM format for textural feature image analysis.

#### SUV Statistics

Vendor software was used for SUV statistics calculation. SUV values were derived from the radioactivity concentration in the tissue, the dose of radioactivity administered and the patient’s weight. All SUV values were corrected for a patient’s body weight. Maximum, mean, and peak SUV values were calculated inside each delineated lesion (SUV_max_, SUV_mean_, SUV_peak,_ respectively) (**Supplementary Materials 1**). Each lesion VOI was copied on the contralateral breast, and the corresponding SUV values subtracted for background correction. The percentage variation was then calculated between FLT1 and FLT2 for all SUV statistics.

#### Texture Analysis

Feature extraction was performed with the open-source S-IBEX software, implemented in MatLab environment (Math-Works, Boston, MA, USA) (18) and IBSI compliant (19, 20). Voxels were resampled to 1 × 1 × 1 mm^3^ size to take into account the different acquisition parameters of the image sample. A total of 148 features were extracted: 25 first order features (*i.e.* based on SUV histogram, namely Voxel statistics), 28 morphological features, and 95 second or higher orders (**Supplementary Materials 1**). Gray level quantization was fixed to 32 bins for second order features and IVH (Intensity Volume Histogram) features.

### Response Evaluation

#### Radiological PET Response

The PET response to therapy was determined according to EORTC 1999 criteria (21) between FLT1 and FLT2.

#### Pathological Response Evaluation

At the end of the chemotherapy course, all patients underwent surgery (**Table 1**). Histopathological analysis was performed and details are reported in **Supplementary Materials 2**. To evaluate the pathological response based on histopathological findings, we opted for the web MD Anderson Residual Cancer Burden (RCB) calculator (22, 23). This software enabled us to identify four different categories of RCB: RCB-0, RCB-I, RCB-II, and RCB-III corresponding to complete pathological response, presence of minimal residual disease (almost complete response), presence of moderate residual disease, and presence of extensive residual disease, respectively. We classified pathological responses as complete (pCR) for both RCB-0 and RCB-I, and partial (pPR) for both RCB-II and RCB-III.

**Table 1:** Main patient characteristics.

ID	Age [years]	cTNM^1^	Surgery^2^	Histological type^3^	ER, PgR, MIB-1^4^	ypTNM^5^	PET/CT scanner^6^
1	60	T2 N1	TM+ALND	IDC	ER 0%	T0 N0	D LS
					PgR 0%		
					MIB-1 40%		
2	73	T2 N0	QUAD+SND	IDC	ER 95%	T1c N0	B mCT
					PgR 90%		
					MIB-1 10%		
3	58	T2 N0	TM+SND	IDC	ER 0%	T0 N0	B mCT
					PgR 0%		
					MIB-1 25%		
4	71	T2 N0	TM+ALND	IDC	ER 95%	T1b N0	D LS
					PgR 18%		
					MIB-1 18%		
5	53	T2 N1	TM+ALND	IDC	ER 90%	T1b N1mic	D LS
					PgR 5%		
					MIB-1 45%		
6	61	T2 N0	QUAD+ALND	IDC	ER9 100%	T1 N1	B mCT
					PgR 5%		
					MIB-1 5%		
7	53	T4 N1	TM+ALND	IDC	ER 100%	T0 N3a	D LS
					PgR 100%		
					MIB-1 35%		
8	31	T2 N1	TM+ALND	IDC	ER 80%	T1a N0	D LS
					PgR 15%		
					MIB-1 35%		
9	59	T4d N3a	TM+ALND	IDC	ER 0%	T4dN0	D LS
					PgR 0%		
					MIB-1 40%		
10	61	T2 N0	TM+SND	IDC	ER 95%	T0 N0	D LS
					PgR 2%		
					MIB-1 15%		
11	44	T1c N1	QUAD+ALND	IDC	ER3 0%	T1a N0	D LS
					PgR 0%		
					MIB-1 5%		
12	44	T4b N0	TM+ALND	IDC	ER 0%	T0 N0	B mCT
					PgR 0%		
					MIB-1 25%		
13	55	T2 N1	QUAD+ALND	IDC	ER 0%	T0 N0	B mCT
					PgR 0%		
					MIB-1 40%		
14.	47	T1c N1	QUAD+SND	IDC	ER 95%	Tis N0	D LS
					PgR 0%		
					MIB-1 35%		
15	54	T2 N0	QUAD+SND	IDC	ER 60%	T0 N0	D LS
					PgR 3%		
					MIB-1 23%		

^1^cTNM, cytological TNM classification.

^2^QUAD, quadrantectomy; ALND, axillary lymph node dissection; SND, sentinel node dissection; TM, total mastectomy.

^3^IDC, Invasive ductal carcinoma.

^4^ER, estrogen receptor; PgR, progesterone receptor; MIB-1, proliferation index.

^5^ypTNM, post-therapy pathological TNM classification.

^6^D LS, Discovery LS PET/CT scanner; B mCT, Biograph 20 mCT PET/CT scanner.

### Statistical Analysis

Continuous data corresponding to SUV statistics and the 148 texture features were considered as percentage changes between FLT1 values and FLT2 values with respect to FLT1: (FLT1−FLT2)/FLT1*100. This has a threefold purpose: 1) it reflects the reasoning of the clinician who is accustomed to evaluating changes from baseline (*i.e.* FLT1); 2) it reflects the longitudinal nature of the study; and 3) the use of percentage changes in place of absolute values allows the comparison of results obtained with different scanners.

In order to identify the most robust features, the intraclass correlation coefficient (ICC) was computed between the four contours of each lesion obtained with expansion and contraction. This analysis was performed on SUV statistics and texture features and also on their percentage changes. The two-way random effects model (24) was used for ICC calculation. The selection of features least impacted by contouring variability was based on the lower confidence interval of ICC and a threshold of ≥0.60 was used as the cutoff value (25).

The accordance between PET response and MD Anderson criteria was tested with the Fisher exact test.

Both the radiological PET response and the pathological treatment response were logistically modeled. LASSO variable selection was used to identify the variables most capable of classifying response correctly (*i.e.*, Least Absolute Shrinkage and Selection Operator *L*_1_ penalized regression) (26). A leave-one-out cross-validation (LOOCV) procedure allowed us to fine-tune the LASSO complexity parameter. The choice of LASSO over ridge regression (*i.e.*, *L*_2_ penalized regression) or elastic-net (*i.e.* a compromise between the previous two) was motivated by the need to sensibly reduce the number of covariates in the model due to complexity. The ability of SUV statistics and textural features to describe the PET response was first investigated. Then, the possibility of describing the pathological therapy response (MD Anderson index) based on SUV statistics, textural features, and PET response was evaluated. With respect to model calculation, complete response (CR and pCR) was considered equal to 1, while partial response (PR and pPR) were considered equal to 0.

Statistical analyses were carried out with R 4.0.4 (27) and package glmnet 4.1-1 (28) adopted for regularized regression analysis.

## Results

Of the 20 patients selected for the study, three did not have available histology data (one patient refused surgery and two patients were not operated because of systemic disease progression). Two patients did not undergo the second FLT PET/CT scan and were therefore not included in the analysis. Thus, a total of 15 patients were evaluable. Median age at baseline was 54 years with a 10.5 year interquartile range (**Table 1**). Ten patients underwent image acquisition on the Discovery LS scanner, and the remaining five on the Biograph 20 mCT scanner. Given that lymph nodes were positive in 5/15 patients, only primary lesions were considered for the analysis.

On the basis of MD Anderson criteria, there was a complete absence of neoplastic disease (pCR) classified as RCB-0 in eight patients and RCB-I in one patient. The remaining six patients showed a partial remission (pPR) classified as RBC-II in five patients and RCB-III in one patient.

On the basis of EORTC 1999 criteria, a comparison of PET images revealed a complete response (CR) to treatment in seven patients and a partial response (PR) in eight patients, with an average reduction in the SUV_max_ value of 44%.

**Figure 2:**
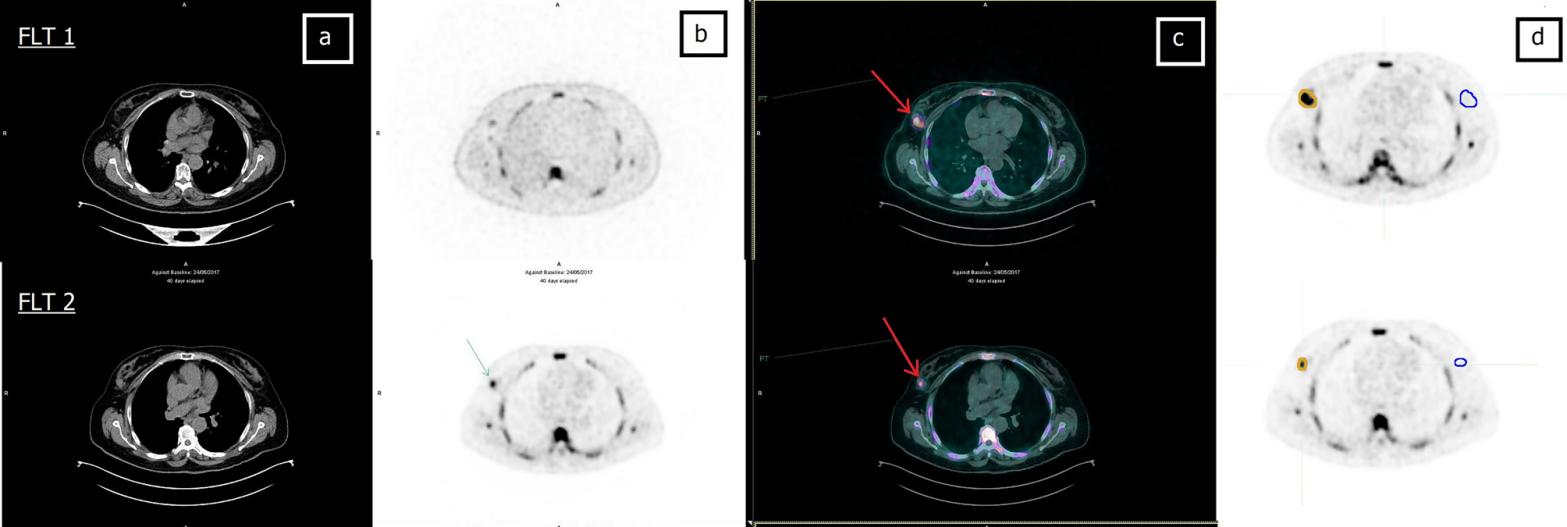
Sixty-one-year-old patient (patient 6 in **Table 2**) undergoing NCT for locally advanced right BC. **(A)** CT, **(B)** FLT-PET, **(C)** CT and FLT-PET fusion, and **(D)** delineation of lesion (yellow line) and controlateral breast (blue line). Upper row: FLT 1; lesion SUV_max _= 9.1. Bottom row: FLT 2; lesion SUV_max_ = 5.6. Red arrow indicates breast lesion. Post mastectomy histological examination revealed the absence of neoplastic tissue. EORTC PET classification: PR (partial remission).

**Figure 3:**
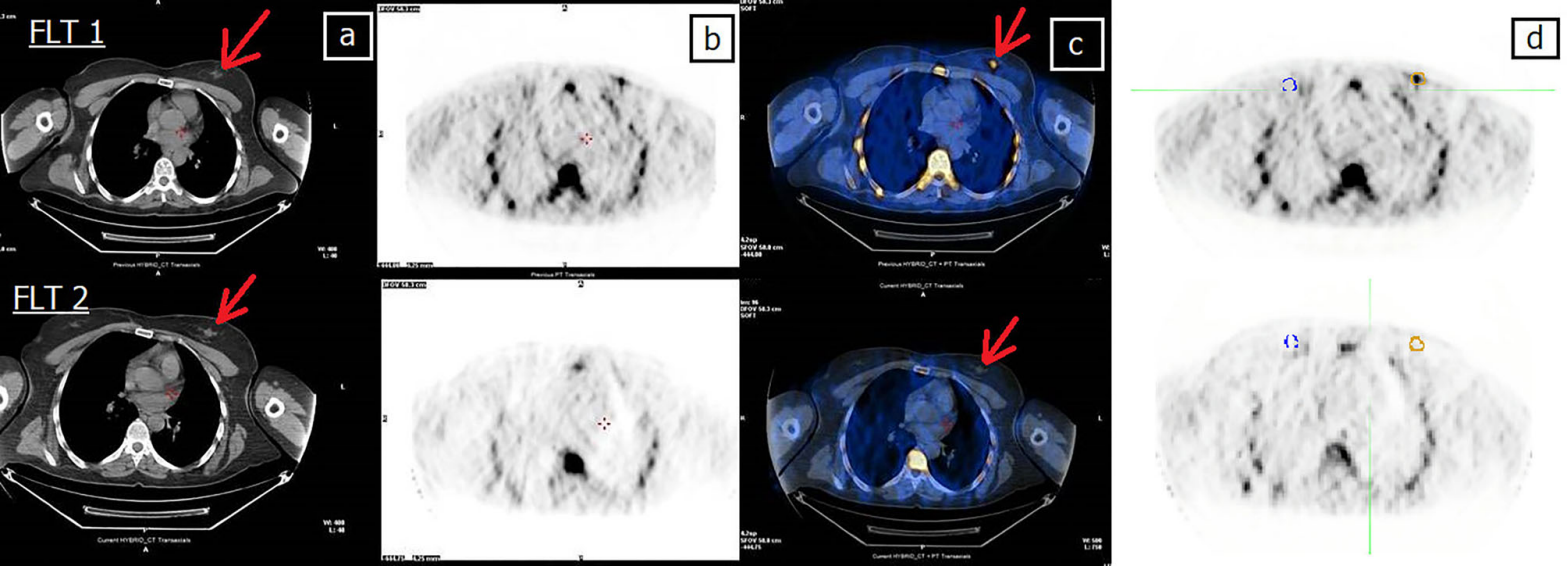
Forty-four-year-old patient (patient 11 in **Table 2**) undergoing NCT for locally advanced right BC. **(A)** CT, **(B)** FLT-PET, **(C)** CT and FLT-PET fusion, and **(D)** delineation of lesion (yellow line) and controlateral breast (blue line). Upper row: FLT 1; lesion SUV_max _= 2.7. Bottom row: FLT 2; FLT 2 shows the complete disappearance of uptake in the tumor area, still evident at CT (arrow). Red arrow indicates breast lesion. Post mastectomy histological examination revealed the absence of neoplastic tissue. EORTC PET classification: CR (complete remission).

Comparative analysis of the results showed that PET images were consistent with the subsequent histological examination in 13 (87%) patients (seven with pCR and six with pPR) (**Table 2**). Examples of patients with a PET PR and PET CR to treatment are shown in **Figures 2** and **3**, respectively.

**Table 2:** Comparison of the results of PET [EORTC (21)] and MD Anderson [RCB pathological response (22, 23)] criteria.

		MD criteria	
		pCR	pPR
PET response	CR	7	0
	PR	2	6

CR, complete response; PR, partial response.

A discrepancy was observed between PET results and histological findings in two patients (**Table 2**), both of whom showed a PET PR but a CR at pathological examination (pCR, one RCB-0 and one RCB-I). The Fisher exact test revealed a significant (p = 0.007) association between PET response and MD Anderson criteria.

Among 3 SUV statistics and 148 textural features, all 3 SUV statistics and 39 textural features were classified as robust against contour delineation (**Table 3**). These indices were used for LASSO logistic analysis. The analysis performed on each original data set (FLT1 or FLT 2) showed a lower number of features robust against contour variability (**Supplementary Material 3**).

**Table 3:** SUV statistics and robustness of textural features robust against contour delineation.

	ICC	ICC lower bound
SUV_max	0.99	0.98
SUV_peak	0.99	0.97
SUV_mean	0.98	0.96
ID_Variance	0.98	0.97
ID_Median	0.94	0.87
ID_Min	0.80	0.64
ID_10thPercentile	0.92	0.83
ID_90thPercentile	0.97	0.94
ID_InterquartileRange	0.89	0.78
ID_Range	0.99	0.97
ID_MeanAbsoluteDeviation	0.96	0.92
ID_RobustMeanAbsoluteDeviation	0.94	0.88
ID_MedianAbsoluteDeviation	0.96	0.92
ID_CoefficientOfVariation	0.94	0.88
ID_QuartileCoefficientOfDispersion	0.81	0.64
ID_Energy	0.97	0.92
ID_RootMeanSquare	0.97	0.94
IVH_VolumeIntFract_90	0.91	0.81
IVH_IntensityVolFract_10	0.79	0.62
IVH_AreaUnderIVHCurve	0.80	0.62
LIF_LocalIntensityPeak	0.98	0.96
GLCM_222_1JointAverage	0.81	0.65
GLCM_222_1SumAverage	0.81	0.65
GLCM_222_1AutoCorrelation	0.81	0.64
GLCM_222_1InformationMeasureCor1	0.78	0.60
GLDZM_GLNonuniformity	0.85	0.71
GLDZM_ZDNonuniformity	0.82	0.65
GLRLM_ShortRunEmphasis	0.78	0.60
GLRLM_LongRunEmphasis	0.80	0.63
GLRLM_HighGLRunEmpha	0.81	0.64
GLRLM_ShortRunHighGLEmpha	0.80	0.63
GLRLM_LongRunHighGLEmpha	0.83	0.67
GLRLM_GLNonuniformity	0.90	0.79
GLRLM_RLNonuniformity	0.95	0.87
GLRLM_RunPercentage	0.81	0.64
GLSZM_LargeZoneHighGLEmpha	0.90	0.80
GLSZM_GLNonuniformity	0.85	0.71
NGLD_HighGLCountEmpha	0.81	0.65
NGLD_HighDepenHighFLEmpha	0.78	0.60
NGLD_GLNonuniformity	0.89	0.78
NGLD_DepCountNonuniformity	0.84	0.69
NID_Coarseness	0.81	0.64

Intraclass correlation coefficient (ICC) calculated using the two-way random effects model. Assumption: absolute agreement. Thresholds: lower bound of 95% CI of ICC and a threshold of 0.60 for ICC (24, 25) (good reliability).

**Table 4** shows the LOOCV-estimated LASSO logistic model of the binary radiological PET response. In addition to SUV_max_, the model selects IVH_VolumeIntFract_90 as an important texture feature for classification. The selected textural feature stands for “Volume at Intensity Fraction 90%” [IBSI feature classification: BC2M (18)]. This feature belongs to the “Intensity Volume Histogram” feature set [IBSI family classification: P88C (18)] and describes the relationship between discretized intensities and the fraction of volume containing at least one determined intensity value (18). IVH_VolumeIntFract_90 measures the largest volume fraction that has a normalized intensity of at least 90% (19). The model showed that a decrease in SUV_max_ at FLT2 with respect to baseline was more likely to lead to a CR. Conversely, an increase in IVH_VolumeIntFract_90 led directly to a higher probability of observing a CR. Additional models are reported in **Supplementary Materials 4**.

**Table 4:** Coefficients, tuning parameters, and classification error rate for the two LASSO logistic models for radiological PET response (26).

Coefficients	
Intercept	−3.316
SUV_max_	0.041
IVH_VolumeIntFract_90	−0.005
λ_min_	0.120
*Classification Error rate*	*0.000*

The model allows the algorithm to select the covariates to be included in the model without constraints.

**Table 5** shows two LOOCV-estimated LASSO logistic models of the post-surgery binary pathological response (MD Anderson index). The first model was fitted without including PET response as covariate. No linear combination of any subset of the covariates (SUV statistics and texture features) is considered useful for classifying the MD Anderson response. In contrast, the second model was fitted to include the PET response as covariate. The inclusion of PET response among the radiomic covariates (SUV statistics and texture features) resulted in LASSO selecting a combination of variables that were informative about MD Anderson response. Despite the strong association between PET response and MD Anderson index (**Table 2**), LASSO also selected texture features as informative about MD Anderson classification. The two selected radiomics features are ID_Range and ID_CoefficientOfVariation, which stand for “Intensity-based Range” [IBSI feature classification: 2OJQ (18)] and “Intensity-based Coefficient of Variation” [IBSI feature classification: 7TET (18)], respectively. Both features belong to the “Intensity-based” features set [IBSI family classification: UHIW (18)] which describes how intensity values are distributed within the VOI. In particular, ID_CoefficientOfVariation measures the dispersion of intensity values inside the VOI, while ID_Range is defined as the difference between the maximum and minimum value. The model showed that a decrease in ID_Range or ID_CoefficientOfVariation at FLT2 with respect to the baseline value or a PET response equal to CR was more likely to lead to a pCR.

**Table 5:** Coefficients, tuning parameters, and classification error rate for the two LASSO logistic models for pathological response (MD Anderson criteria) (26).

Coefficients	without	with
	PET response	PET response
	as covariate	as covariate
Intercept	0.405	−1.842
PET response	–	1.313
ID_Range	–	0.006
ID_CoefficientOfVariation	–	0.001
λ_min_	0.332	0.204
*Classification Error rate*	*0.400*	*0.133*

The PET response is added to the second model as covariate. Both models allow the algorithm to select the covariates to be included in the model without constraints.

## Discussion

The potential value of PET in monitoring response to chemotherapy in breast cancer has yet to be confirmed. The majority of studies to date have been conducted using PET with FDG, while only a few have focused on PET with FLT, mainly in heterogeneous series of patients at different stages of disease, undergoing different chemotherapy regimens and, in particular, with different timing than chemotherapy.

The present study, carried out in a population treated with the same NCT schedule (*i.e.* six cycles each) showed that an early PET with FLT, performed after the first cycle of therapy, was able to classify the pathological response in 100% of cases. It is worthy of note that, despite the limited number of patients analyzed, treatment modality and schedules were homogeneous.

The use of FLT PET/CT scans in breast cancer management has been described in several studies with small cohorts of patients. In a pilot study of 14 patients, Pio et al. (29) reported that a reduction in mean FLT uptake in primary and metastatic tumors after the first course of chemotherapy was significantly correlated with late tumor marker levels and tumor size. Kenny et al. (30) showed that changes in FLT uptake within the first week of chemotherapy in 13 patients with stage II-IV BC were detectable in FLT images. Their results highlighted that the reduction in SUV uptake observed in 27 lesions on FLT images preceded changes in tumor size and was able to discriminate between clinical response (six patients) and stable disease (six patients) (p-value = 0.022, Mann-Whitney test). In addition, the authors also showed that FLT images could be performed with high reproducibility (repeated FLT image acquisition with a two- to 10-day time interval, p-value = 0.95 Wilcoxon signed test).

The majority of studies in the literature are based on maximum SUV uptake. Although different statistical indices have been proposed to describe the maximum uptake [*e.g.* SUV_max_, SUV_peak_, SUV_95th_ (31)], they are not able to detail the non-uniform uptake distribution of the tracer within the lesions and its variation during therapy. Thus, the information provided by these classic indices may be incomplete. It is well known (10) that lesions can be characterized by heterogeneity of tracer distribution in relation to both cellularity and vascularization, hypoxia, or necrosis. For this reason, the estimate of maximum SUV may not faithfully represent the changes related to the effects of chemotherapy.

Radiomics, the process of computerized extraction of functions from radiographic images, is a new strategy for highlighting subtle changes in the tumor region that works by quantifying the sub-visual patterns that may escape human identification. In a recent review, Sollini et al. (31) evaluated the role of PET radiomics in breast cancer, focusing in particular on methodological aspects. Their analysis highlighted significant heterogeneity in published studies in relation to the acquisition, reconstruction, segmentation, and processing of radiomics, suggesting that much of the current evidence on the clinical role of radiomics is only available at a feasibility level. Textural feature extraction has also been tested on FLT images of other tumor types. Dehdashti et al. (32) analyzed FLT images acquired for 13 patients with advanced colorectal cancer before and 2 weeks after the start of neoadjuvant chemotherapy. The authors showed that, during-therapy, low FLT uptake (SUV_max_ < 2.2) and high percentage change in FLT uptake (60%) were predictive of improved disease-free-survival (p < 0.05 for both values). They also found that pre-therapy FLT uptake was not a significant predictor of outcome and did not correlate with disease-free-survival. Ulrich et al. (33) exploited the usefulness of radiomics textural feature extraction on FLT images for patients with head-and-neck cancer. Thirty patients with advanced-stage oropharyngeal or laryngeal cancer treated with definitive chemoradiotherapy were included in the study. The authors found that smaller and more homogenous lesions (described by different textural feature indices) at baseline were associated with better prognosis (p-value < 0.05).

In our study, we combined both SUV statistics and radiomic features. The LASSO logistic regression selects the most informative features of the dataset for classification. The usefulness of IVH_VolumeIntFract_90 is, thus, implicit in its selection because otherwise LASSO would have selected only a combination of SUV statistics. Nonetheless, we do not have a separate test set to evaluate the model performance.

Our models show that the information capable of describing the PET response to treatment is localized in the upper part of the SUV histogram, but is not fully reflected by the SUV_max_. In fact, the models also include the information provided by the textural feature IVH_VolumeIntFract_90. Our findings are in agreement with those of Baiocco et al. (34) who found that the SUV_95th_ (*i.e.* median computed on the upper 10% of the SUV distribution) was a more robust index than SUV_max_ value for uptake characterization. The present study, and the results reported by Baiocco et al. (34), confirms the role of the upper part of the SUV distribution and highlight the need to define new indices capable of overcoming the limits of classic SUV statistics. In fact, as suggested by Baiocco et al. (34), the single voxel count SUV_max_ normally represents an outlier of the SUV histogram.

With regard to the ability to describe the post-surgery pathological response, the LASSO model selected both the PET radiological response and two textural features as most representative of therapy response. The inclusion of two textural features highlights that EORTC criteria alone are inadequate to interpret pathological response and that additional information is needed to correctly asses treatment response based on early imaging. The selection of textural features in both models highlighted the role played by advanced imaging indices in describing the response to treatment. However, the obtained results can only be considered descriptive of the considered patient population, and further investigation in different and larger case series is needed to confirm the predictive power of the model.

Data in the literature have shown that magnetic resonance imaging (MRI), alone or associated with FDG PET, could represent a non-invasive technique for monitoring response to NCT and for assessing residual disease. In particular, in a single-center study of 93 patients with breast cancer treated with NCT, Pengel et al. (35) reported that FDG PET and MRI had a complementary predictive ability. Using FDG PET (SUV_max_ relative reduction) and MRI (relative change in largest tumor diameter) together in a multivariate analysis combined with breast cancer subtypes, the area under the curve (AUC) was 0.90 (95% CI: 0.83–0.96) (30). The AUCs of single imaging modality were 0.78 (95% CI: 0.68–088) for FDG PET and 0.79 (95% CI: 0.70–0.89) for MRI (36). The association of MRI images and FLT PET/CT may therefore provide further information on response to NCT in breast cancer patients.

A limitation of our study was the intrinsic difference between images in terms of both scanner and parameter acquisition (*i.e.* slice thickness, voxel dimension). It is known that, especially for textural features, these factors may influence the statistical analysis and reduce the robustness of extracted textural feature indices (35). In addition, the starting point of each patient may be different in terms of both maximum uptake and distribution inside the lesion. Given that our clinical interest focused on uptake variation as a surrogate of clinical response and that the absolute value of textural features may be influenced by different scanner acquisition and image characteristics, we chose to use the percentage variation of textural features rather than absolute values. This choice may have overcome the loss in textural feature robustness due to image acquisition parameters. This was also confirmed by the robustness analysis performed on our data, where the data calculated as percentage difference between FLT1 and FLT2 was more robust than the data directly extrapolated from single images (**Table 3** and **Supplementary Materials 3**). A larger patient cohort is needed to properly investigate this point.

Another weakness of our study was the impossibility of evaluating prediction efficacy due to the small sample size involved. In fact, our results, despite their fairly good classification capability, can only be used to describe the current patient set. Indeed, it must be taken into account that the analyzed population was a subgroup of patients extracted from a phase II trial. Thus, a new study with an extended patient cohort would permit the assessment of the prediction capacity of the model in a different population.

## Conclusions

The reliability of a FLT-PET textural feature approach for the correct and early prediction of response to treatment has yet to be clarified. The choice of the most accurate parameters represents the main problem preventing its routine and generalized use. Our study suggests the potential usefulness of FLT-PET textural feature for early monitoring of NCT response. In particular, the data deriving from radiomics analyses, more informative than those of the semi-quantitative SUV histogram parameters, reinforce the idea that textural feature may be predictive of response to treatment. Further studies on larger populations are warranted to confirm the role of FLT-PET as a tool to tailor therapy, reducing the risk of exposing unresponsive patients to unnecessary and harmful cycles of chemotherapy

## Data Availability Statement

The datasets presented in this study can be found in online repositories. The names of the repository/repositories and accession number(s) can be found in the article/**Supplementary Material**.

## Ethics Statement

The studies involving human participants were reviewed and approved by Comitato Etico della Romagna, Eudract number: 2014-002602-20; Protocol Code: IRST174.09. The patients/participants provided their written informed consent to participate in this study.

## Author Contributions

Conceptualization: LF, FM, IA, MB, and AS. Methodology: IA, MB, and EL. Formal analysis and investigation: IA, EL, MB, LF, FM, EM, and SA. Writing—original draft preparation: MB, IA, LF, FM, and EL. Writing—review and editing: GP, FM, and SA. Supervision: AS, GP, AF, FM. All authors contributed to the article and approved the submitted version.

## Conflict of Interest

The authors declare that the research was conducted in the absence of any commercial or financial relationships that could be construed as a potential conflict of interest.
