# Supplementary material for: Exploratory Analysis of 18F-3’-deoxy-3’-fluorothymidine (18F-FLT) PET/CT-Based Radiomics for the Early Evaluation of Response to Neoadjuvant Chemotherapy in Patients With Locally Advanced Breast Cancer
Source: Front Oncol. 2021 Jun 24;11:601053. doi: 10.3389/fonc.2021.601053 (PMC8264651; doi:10.3389/fonc.2021.601053)
Supplement: Supplementary file 2 [file DataSheet_2.docx]

**Supplementary materials 2**

**Tissue sampling, immunohistochemistry, fluorescent in situ hybridization and pathological response.**

Tumor samples obtained during biopsy were fixed in 10% neutral buffered formalin, embedded in paraffin and were stained with hematoxylin and eosin (EE), for routine histology. Immunohistochemical (IHC) staining was performed using an automated immunostainer (Ventana); ER (clone SP1), PgR (clone 1E2), Ki67 (clone MIB1), HER2 (clone 4B5) antibodies were used. Biomarker positivity was detected and semi-quantitatively quantified as the percentage between immunopositive tumor cells and the total number of tumor cells. Tumors were considered ER-positive and PgR positive when ≥1% of immunoreactive cells were detected. Ki67 was defined as high when the fraction of positively stained cells was ≥20%, and low when < 20%. Fluorescent in situ hybridization (FISH) was used in first line to determine HER2 status; it was conducted using a dual HER2/Cep17 probe (Path Vysion HER2 DNA Probe kit; Abbott Molecular, Inc., Des Plaines, IL, USA). The gene amplification was considered amplified if the HER2 gene to chromosome 17 centromere ratio was ≥ 2 or if the average HER2 gene copy number per cell was ≥ 6. In cases of “equivocal” HER2 in FISH (average HER2 copy number ≥ 4.0 and < 6.0 signals/cell), reflex test for HER2 was assessed using the alternative IHC HercepTest (DAKO Corporation) which measures the percentage of immunoreactive neoplastic cells defined according to the intensity and completeness of membrane staining and using the 0–3+ recommended scale on the basis of the ASCO-CAP guideline 2013 [20, 21].
