# Supplementary material for: Exploratory Analysis of 18F-3’-deoxy-3’-fluorothymidine (18F-FLT) PET/CT-Based Radiomics for the Early Evaluation of Response to Neoadjuvant Chemotherapy in Patients With Locally Advanced Breast Cancer
Source: Front Oncol. 2021 Jun 24;11:601053. doi: 10.3389/fonc.2021.601053 (PMC8264651; doi:10.3389/fonc.2021.601053)
Supplement: Supplementary file 3 [file DataSheet_3.docx]

**Supplementary materials 3**

Statistical analysis of textural feature robustness (absolute agreement) on data directly extracted from FLT1 and FLT2.

|  | ICC | ICC  lower bound |
| --- | --- | --- |
| SUV_max | 1.00 | 1.00 |
| SUV_peak | 1.00 | 1.00 |
| SUV_mean | 0.90 | 0.77 |
| ID_Variance | 0.99 | 0.98 |
| ID_Median | 0.85 | 0.68 |
| ID_90thPercentile | 0.97 | 0.93 |
| ID_InterquartileRange | 0.98 | 0.97 |
| ID_Range | 0.99 | 0.97 |
| ID_MeanAbsoluteDeviation | 0.99 | 0.99 |
| ID_RobustMeanAbsoluteDeviation | 0.99 | 0.98 |
| ID_MedianAbsoluteDeviation | 0.99 | 0.99 |
| ID_Energy | 0.98 | 0.94 |
| ID_RootMeanSquare | 0.93 | 0.83 |
| LIF_LocalIntensityPeak | 1.00 | 1.00 |
| GLCM_222_1SumVariance | 0.78 | 0.66 |
| GLCM_222_1CLusterTendendcy | 0.78 | 0.66 |
| GLCM_222_1ClusterProminence | 0.74 | 0.61 |
| GLSZM_LargeZoneHighGLEmpha | 0.85 | 0.72 |
| NGLD_HighDepenLowGLEmpha | 0.8 | 0.67 |
| NGLD_HighDepenHighFLEmpha | 0.79 | 0.67 |
| NGLD_DepCountVariance | 0.85 | 0.68 |
