# Supplementary material for: Exploratory Analysis of 18F-3’-deoxy-3’-fluorothymidine (18F-FLT) PET/CT-Based Radiomics for the Early Evaluation of Response to Neoadjuvant Chemotherapy in Patients With Locally Advanced Breast Cancer
Source: Front Oncol. 2021 Jun 24;11:601053. doi: 10.3389/fonc.2021.601053 (PMC8264651; doi:10.3389/fonc.2021.601053)
Supplement: Supplementary file 4 [file DataSheet_4.docx]

**Supplementary materials 4**

Additional LASSO models are reported here for completeness.

LASSO on PET response

| coefficients | with  constraint on SUV_peak_ | with  constraint on SUV_mean_ |
| --- | --- | --- |
| intercept | -6.817 | -9.696 |
| SUV_peak_ | 0.089 | ─ |
| SUV_mean_ | ─ | 0.120 |
| IVH_VolumeIntFract_90 | -0.011 | -0.003 |
| λ_min_ | 0.120 | 0.219 |
| *classification*  *error rate* | *0.000* | *0.20* |

Note that the IVH_VolumeIntFract_90 index is selected by the LASSO model even if a constraint is added on some SUV statistics (SUVpeak and SUVmean).
